# Identifying Avoidable Emergency Department Visits and the Potential Role of Community Health Centers in Northern Italy: A Retrospective Observational Study

**DOI:** 10.1002/hcs2.70103

**Published:** 2026-09-25

**Authors:** Silvia Caristia, Isabella Santomauro, Erika Bassi, Laura Ferraris, Alberto Dal Molin, Fabrizio Faggiano

**Affiliations:** ^1^ Department for Sustainable Research and Ecological Transition University of Piemonte Orientale Vercelli Italy; ^2^ Department of Translational Medicine University of Piemonte Orientale Novara Italy; ^3^ Emergency Department, S.S. Trinità Hospital Borgomanero Italy

**Keywords:** emergency avoidable admission, emergency department, nursing, primary health care, retrospective observational study

## Abstract

**Background:**

Emergency departments (EDs) are experiencing a growing burden of non‐urgent presentations, which frequently signal deficiencies in access to primary and community‐based healthcare. Evidence suggests that a substantial proportion of ED visits could be safely and effectively managed within community care settings. The general aim of the study was to identify which ED visits could have been managed within primary and community care services in a scenario of full implementation of community health centers.

**Methods:**

A retrospective observational study was conducted. This study examined data from the first‐level ED at S.S. Trinità Hospital, Borgomanero in Northern Italy. It involved analyzing all access data from the ED in 2022. Avoidable ED visits were identified using triage assessments, multidisciplinary team, double‐blinding nursing assessments, and data validation.

**Results:**

Of the 43,437 ED visits in 2022, 91.8% were nonurgent (*n* = 39,864). We identified 46.3% (*n* = 20,104) of the ED visits as avoidable, with the most common characteristics of presentation being trauma or burns, fever, eye issues, urological symptoms, ear nose and throat problems, lower back pain, and swelling.

**Conclusions:**

Our findings highlight the need to strengthen primary care services through cohesive, accessible, and timely interprofessional networks to effectively address non‐urgent healthcare needs and reduce the burden on ED settings.

## Graphical abstract

Using data from an Italian emergency department (ED), ED visits preventable through integrated community care models (community health centers) were identified. ED visits were classified based on presenting symptoms, comorbidities, medication use, and age. The results underscore the necessity of strengthening primary care to reduce hospital overcrowding.

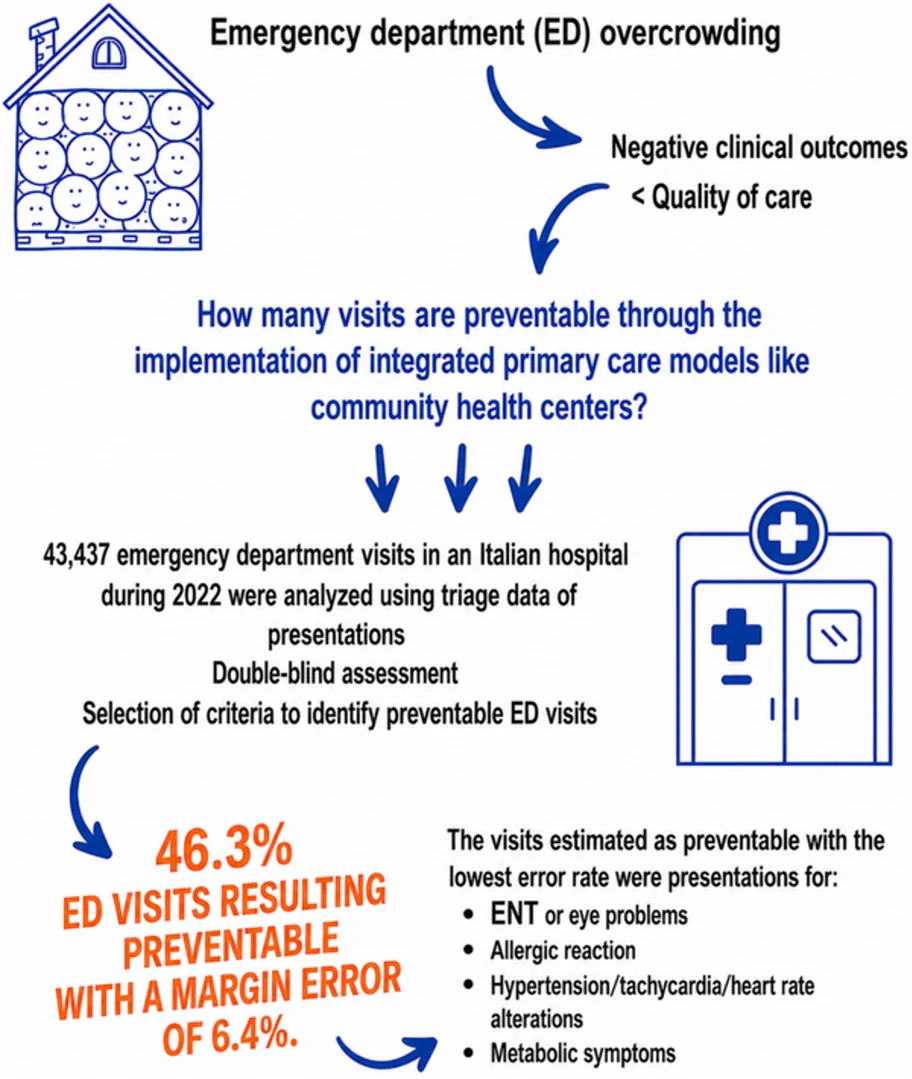

**Abbreviations**
CHC: community health center
CI: confidence interval
COPD: chronic obstructive pulmonary disease
ED: emergency department
ENT: ear, nose, and throat
FCN: family and community nurse
GP: general practitioner
LHU: local health unit
OR: odds ratio
PC: primary care
SD: standard deviation

## 1 Background

According to the Institute of Medicine, the growing issue of overcrowding in emergency departments (EDs), mainly caused by the ageing of the population, has become a global concern, further intensified by the recent COVID‐19 pandemic [1]. Although there is no standard definition, ED overcrowding can be described as a situation where the functionality of the ED is compromised due to the disproportionate ratio between the healthcare demand–represented by the number of patients awaiting and receiving care—and response, the available human and/or structural resources needed to address it.

In an organizational perspective, focusing on input factors [2], a primary contributor to ED overcrowding is the occurrence of inappropriate visits or, given the lack of a standard definition [3], “potentially avoidable emergency visits” [4]. They are emergency visits that could be managed in a home context or primary care (PC) setting, or that do not require immediate nursing or medical intervention, nor lead to greater comfort or quality of life for the patient [5]. Reducing these visits could significantly lower healthcare expenses and improve both the quality of care and the patients' quality of life [6].

Since the 1990s, several specific conditions have been recognized as ambulatory care‐sensitive, under which a visit is considered potentially avoidable through optimal PC setting [7, 8]. In this regard, Purdy et al. [7] identified 36 potential health conditions, such as abdominal pain caused by constipation, headache, alcohol‐related conditions, and diabetes, that could be managed effectively in PC settings to prevent hospitalization.

The demographic and epidemiologic transition due to the population ageing and the consequent impact on health services, including overcrowding of the EDs, are the main determinants of the transition from health care centered on hospitals, toward a model centered on primary care fostered by the World Health Organization and in progress of implementation in many countries [9, 10], especially after the COVID‐19 pandemic. A pillar of this new model is represented by the community health centers (CHCs), frontline organizations in national PC systems [11]. They have prominent tasks in providing effective, efficient, equal, accessible, and affordable healthcare to local communities [12, 13].

Recent studies on the impact of CHCs showed generally good improvement of quality of care [14], reduction of EDs accesses [15, 16], and better patient outcomes. These services have been recently introduced in the Italian context as well. They are characterized by multi‐professional teams, physicians, family and community nurses (FCNs), and other health care personnel, delivering both primary and specialized care. In this scenario, FCNs, within the CHCs, play a crucial role in managing the input factors of hospital overcrowding, particularly in meeting the clinical and care demands of the population, by activating social and health networks [17, 18].

To date, no studies have identified which ED visits may be avoidable and could be appropriately managed within a PC centered healthcare model based on CHCs.

Therefore, the aim of this study was to identify ED visits that could be managed within primary and community care services in a scenario of full implementation of CHCs, in a local health unit (LHU) in Northern Italy, in which the implementation of these services was still not started.

## 2 Methods

### 2.1 Study Design

This retrospective observational study was conducted by examining data on ED visits from the first‐level ED at S.S. Trinità Hospital, Borgomanero, LHU of Novara, Northern‐Italy.

All ED visits data from January 1 to December 31, 2022, as retrieved from the LHU database, were analyzed.

This paper adhered to Strengthening the Reporting of Observational Studies in Epidemiology reporting guidelines [19].

### 2.2 Setting

The Italian healthcare system provides universal coverage through the National Health Service, delivering publicly funded primary and hospital care. Access to primary care services, including general practitioners and CHCs, as well as ED services, is direct and unrestricted, whereas access to specialized outpatient and hospital services requires physician referral. Although healthcare is predominantly funded through public taxation, patient co‐payments are required for selected services, including non‐urgent ED visits, specialist consultations, and diagnostic tests.

The SS. Trinità Hospital in Borgomanero is the primary specialized hospital care facility for the northern area of the Province of Novara, with 300,000 inhabitants in Northern Italy. It is a secondary‐level (spoke) hospital within a hub‐and‐spoke public healthcare network, providing a broad range of medical, surgical, and emergency services to the local population, while referring patients requiring highly specialized diagnostic or therapeutic interventions to tertiary referral (hub) hospitals. The Borgomanero ED is the only emergency facility in the local area, with 43,000 accesses in 2022. In the area of Borgomanero, the following public health services are available: three CHCs, a drug addiction service, and medical on‐call service. Except for the service continuity of care, which guarantees basic medical care at home 24 h/7 days, all these primary care services were only open daily during working days.

### 2.3 Data

The data extracted from the clinical files of the ED and included in the analysis were: personal information (i.e., age and sex), clinical data (i.e., symptoms and main characteristics of presentation, pre‐existing diseases, drug use, and diagnosis at discharge), ED visits data (i.e., triage color code, hour and day of ED visits, and mode of access) and discharge data (i.e., discharge color code and type of discharge). The availability of a variable identifying the patients allowed to identify repeated ED visits and those occurring 1 week after discharge from the ED.

### 2.4 Methods and Criteria for Categorizing ED Visits

Avoidable ED visits were defined according to the following criteria: (1) white, green, and blue codes assigned by the ED triage, indicating non‐urgent or less urgent cases (orange and red codes were defined non‐deferrable due to urgency; (2) accesses during the day and weekdays; (3) the chief complaint, along with any coexisting symptoms or signs (blood pressure, glycaemia, body temperature, etc.); (4) pre‐existing diseases; and (5) the patient's age.

The identification of avoidable ED visits was carried out through a 3‐step process:

**Step 1:** Preliminary identification. Initially, we identified potentially avoidable ED visits based on the following criteria:

(1) All visits are classified as white code;

(2) Visits classified as green or blue codes, with the exclusion of cases with symptoms that could suggest any severe underlying diseases, like, for example, acute neurological symptoms, chest pain, or abdominal pain and high blood pressure, and/or glycemic spikes in individuals without known pre‐existing conditions (Box 1). These exclusion criteria were submitted to a validation process carried out by a multidisciplinary team including the hospital medical director, two ED nurses with expertise in family and community nursing, and a research fellow affiliated with the study team.

Box 1 **Criteria used to identify avoidable ED visits.**

**Table:** 

Triage code	Code definition	Inclusion criteria	Exclusion criteria
**White code**	**Not urgent:** non‐urgent or of minimal clinical significance	All white codes	None
**Green code**	**Low urgency:** stable condition without evolutionary risk, typically requiring simple mono‐specialist diagnostic and therapeutic interventions	**Steps 1 and 2:** all green and blue codes **Step 3:** green and blue codes with access occurred during CHCs' opening hours (from 8:00 a.m. to 8:00 p.m.)	**Step 1** Accesses for: Abdominal pain Thoracic pain Acute neurological symptoms Other neurological symptoms without pre‐existing neurological diseases Blood pressure rises/heart rhythm alterations without pre‐existing disease (cardiovascular diseases and/or diabetes) Headache without pre‐existing disease (chronic headache and/or hypertension) Glycaemia rise without pre‐existing disease (cardiovascular diseases and/or metabolic disease) or with pregnant women with diabetes Objective dyspnea without pre‐existing COPD or with hypertension Hyperpyrexia Third degree burns Intoxication for substance abuse Syncope **Step 2** Accesses for: Heart rhythm alterations symptoms in association to: dizziness, nausea, dyspnea, breathlessness, tightness in the chest, hypertension (systolic blood pressure > 160 mmHg), syncope, ringing in the ears, and/or fever Obstetric‐gynecological symptoms in pregnant women or women < 42 years old Head trauma in adults and old age persons or trauma in association to amnesia and/or bleeding wound Trauma for accidents in workplace Trauma with bleeding lesion Other neurological symptoms in people ≥ 75 years old Non‐traumatic hemorrhage with hypertension and/or metabolic diseases Amnesia, confusion, or drowsiness Ear, nose, and throat symptoms with nausea and/or dizziness **Step 3** Accesses from 8:01 p.m. to 7:59 a.m.
**Blue code**	**Deferrable urgency:** stable condition without evolutionary risk, with suffering and impact on the general state, typically requiring complex interventions		
**Orange code**	**Urgency:** risk of compromise of vital functions, condition with evolutionary risk or high pain	—	All visits
**Red code**	**Emergency:** interruption or compromise of one or more vital functions	—	All visits

Abbreviations: CHC, community health center; COPD, chronic obstructive pulmonary disease; ED, emergency department; —, not applicable.

**Step 2:** Validation and refinement. A random sample of approximately 5% of the potentially avoidable ED visits (*n* = 1072) was independently reviewed by two ED nurses. This step aimed to refine the initial criteria. The review was based on information extracted from ED medical records, including patient age, sex, presenting symptoms, ED visit characteristics, and known pre‐existing conditions. Discrepancies between the two reviewers were resolved through consultation with an additional panel of physicians and nurses, resulting in a Cohen's kappa coefficient of 0.93. This procedure led to the identification of further exclusion criteria (e.g., specific age thresholds) that had not been considered in the initial selection.

**Step 3:** Final Definition. To the final list of criteria, Steps 1 and 2, a new criterion based on the expected regular opening hours of CHCs (i.e., from 8:00 a.m. to 8:00 p.m. on weekdays) was applied to the ED visits, to estimate the number of avoidable ED visits at the Borgomanero ED during the year 2022.

### 2.5 Variables

All the variables included in the analysis are derived from ED accesses database of the Borgomanero Hospital for the calendar year 2022. They include personal, clinical, ED visit, and discharge‐related that comprise the triage urgency code as well as the discharge code.

### 2.6 Data Analysis

Descriptive analysis was carried out to compare non‐urgent (white, green, and blue codes) ED visits with urgent ones (orange and red). *χ* ^2^ tests (95% of confidence interval [CI]) were used to identify differences based on categorical variables (sex, entry time, day of the week, access mode, characteristics of presentation, and pre‐existing diseases), while differences by age groups were tested using Spearman's *ρ*. Age groups were defined to identify the pediatric population (which in the Italian healthcare system encompasses individuals aged 0–14 years), adults (15–69 years), and older adults (≥ 70 years). The strength of associations was tested using odds ratio (OR), Cramér's *V*, or Spearman's *ρ*. The accuracy of triage assessments was determined by comparing the triage code at access with the discharge urgency code. To identify the characteristics of presentation most likely associated with avoidable ED visits, *χ* ^2^ tests and Spearman's *ρ* were used to conduct a descriptive analysis of avoidable and unavoidable visits across the main characteristics of presentation. To identify the criteria that might be used to estimate avoidable ED visits by the access to CHCs, we first tested the accuracy of the criteria by comparing the type of discharge (home vs. hospitalization/death) for avoidable ED visits. Finally, a sensitivity analysis was carried out by comparing avoidable visits with the outcomes of the access, home discharge, hospitalization, or death, to quantify the error of our set of criteria.

## 3 Results

From January 1 to December 31, 2022, the ED at S.S. Trinità Hospital of Borgomanero registered 43,437 ED visits; the majority were non‐urgent (*n* = 39,864; 91.8%), triaged as green (68.5%), blue (16.5%), or white (6.8%). Most patients were adults aged 15–69 years (56.5%, mean age 48.0, standard deviation [SD] ± 27.5), with no significant difference by sex (females 51.3%). Most ED visits occurred during daytime (72.2%) or on weekdays (71.3%), with patients predominantly arriving at the ED using their own vehicles (75.6%). In the triage evaluation, non‐urgent codes were mainly women (*p* < 0.0001), individuals aged 15–69 years (mean age of non‐urgent codes 46.5 ± 0.1 vs. 65.3 ± 0.4; *p* < 0.0001), accessing during the day and by other means than ambulance (20.2% of non‐urgent codes *vs*. 64.3% of urgent codes arrived by ambulance) (Table 1). In 2022, among the 43,437 ED visits, 85.9% were discharged at home, whereas 13.8% were hospitalized, and 0.3% died (Table 1).

**Table 1:** Characteristics of ED visits at the hospital of Borgomanero in 2022 by non‐urgent and urgent codes (*N* = 43,437).

Variables	Total		Non‐urgent codes		Urgent codes		
			(*n* = 39,864)		(*n* = 3573)		
	*n*	%	*n*	%	*n*	%	*p* a
Sex							< 0.0001
Female	22,298	51.3	20,572	92.3	1726	7.7	
Male	21,139	48.7	19,292	91.3	1,847	8.7	
Age (years)							< 0.0001^b^
< 15	6782	15.6	6,645	98.0	137	2.0	
15–69	24,547	56.5	23,049	93.9	1498	6.1	
≥ 70	12,108	27.9	10,170	84.0	1938	16.0	
Entry time (triage hour)^c^							< 0.0001
Day	31,349	72.2	28,911	92.2	2438	7.7	
Night	12,088	27.8	10,953	90.6	1135	9.4	
Day of entry							0.097
Weekday	30,976	71.3	28,471	91.9	2505	8.1	
Weekend	12,461	28.7	11,393	91.4	1068	8.6	
Access mode							< 0.0001
Ambulances	10,341	23.8	8,043	77.8	2298	22.2	
GP referral	262	0.6	247	94.3	15	5.7	
Other structure	6	0.0	4	66.7	2	33.3	
Own vehicle	32,828	75.6	31,570	96.2	1258	3.8	
Type of discharge							< 0.0001
Discharged	37,320	85.9	35,518	95.2	1802	4.8	
Hospitalized	5992	13.8	4,323	72.1	1669	27.9	
Deceased in ED or during transfer	124	0.3	23	18.5	101	81.5	
Dead	1	0.0	—		—		
Triage code							
White	2959	6.8					
Green	29,741	68.5					
Blue	7164	16.5					
Orange	3062	7.0					
Red	511	1.2					

Abbreviations: CI, confidence interval; ED, emergency department; GP, general practitioner; —, not applicable.

^a^ *χ* ^2^ test with 95% CI.

^b^ Spearman's *ρ*.

^c^ Daytime from 8:00 a.m. to 7:59 p.m., Night from 8:00 p.m. to 7:59 a.m.

Trauma or burn accounted for one‐third of all ED visits (30.0%), followed by abdominal pain (9.4%), obstetrical/gynecological symptoms (7.4%), and fever (7.3%). Most accesses (72.0%) were first‐time presentations to this ED. The more frequent pre‐existing conditions were hypertension (8.7%), cardiovascular diseases (5.5%), endocrine‐metabolic disorder (6.0%), mostly diabetes (3.5%) (Supporting Information S1: Table S1). The diagnoses at discharge included trauma or burn (22.6%), gastrointestinal disorders (9.9%), Cardiovascular events (6.5%), obstetrical/gynecological disorders (6.4%), respiratory events (6.0%), and ear, nose, and throat (ENT) problems (4.6%) (Supporting Information S1: Table S2).

**Figure 1:**
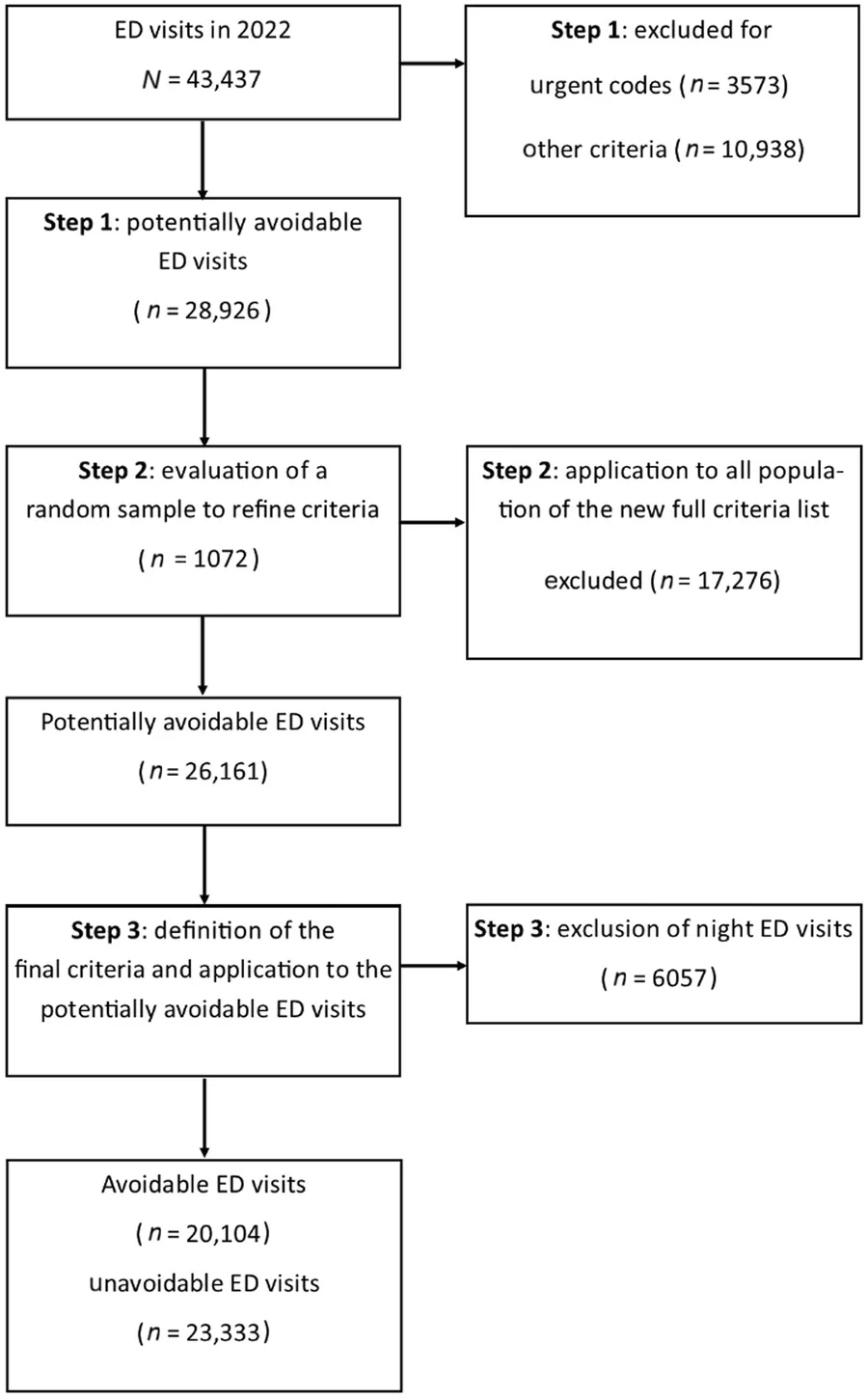
Flow process of the process identification of avoidable ED visits. ED, emergency department.

Figure 1 illustrates the flow process for identifying avoidable ED visits at Borgomanero Hospital in 2022. Out of the total ED visits in 2022, 60.2% (*n* = 26,161) were classified as potentially avoidable ED visits, after excluding visits associated with urgent triage codes and certain cases within the green and blue codes (Steps 1 and 2 of the Material and Methods, Section Methods, 2), such as visits involving chest pain or acute neurological symptoms, identified from the evaluation of the random sample of 1,072 ED visits reviewed by two reviewers (Step 2).

After the exclusion of the cases that arrived in the ED in periods of theoretical closure of the CHC (Step 3), 20,104 visits were classified as avoidable ED visits that could be managed within primary and community care services in a scenario of full implementation of CHCs, accounting for 46.3% of all ED visits (*N* = 43,437) (Figure 1).

Avoidable ED visits were compared with non‐avoidable, by discharge type to validate the proposed criteria, leading to an error rate of 6.4%, representing avoidable ED visits that resulted in hospitalization or death (i.e., negative outcomes), as detailed in Supporting Information S1: Table S3. An analysis of the classification error (*n* = 1,291; 6.4%) revealed that the highest error rates—defined as the proportion of avoidable ED visits resulting in subsequent hospitalization or death—were observed for visits related to social problems (40.8%), referrals for medical tests (31.7%), and psychomotor agitation (25.0%). Almost all of these visits were not categorized as urgent upon discharge, except for four referrals and one case of psychomotor agitation. The incidence of these issues differed significantly between avoidable ED visits ending in home discharge and those resulting in hospitalization or death. In contrast, most avoidable ED visits for hypertension, tachycardia/heart rate alterations, or metabolic symptoms ended in home discharge (Supporting Information S1: Table S4). Patients with avoidable ED visits followed by home discharge were more likely to be aged 15–69 years and to have arrived by private vehicle than patients who were subsequently hospitalized or died (Supporting Information S1: Table S4). Regarding pre‐existing conditions, most were statistically associated with the type of discharge, with the strongest associations observed for heart failure, obesity, chronic obstructive pulmonary disease, urinary and kidney diseases, and hematologic disorders (OR > 3) (Supporting Information S1: Table S4).

Table 2 compares avoidable ED visits to those that were not. Avoidable ED visits were more common among males (*p* < 0.0001) and individuals under the age of 15 (*p* < 0.0001), although the association with age was weak (*ρ* = −0.09). A stronger association was observed with the mode of access (OR = 0.17, *p* < 0.0001), with a higher proportion of individuals arriving by own vehicle in cases where avoidable ED visits (51.0%), whereas ED visits via ambulance were mainly unavoidable (68.7%) (Table 2). Considering pre‐existing diseases, ED visits for patients with pre‐existing conditions were largely unavoidable, particularly for those with cardiovascular or respiratory diseases. Among these, the strongest association emerged for ED visits by patients with pre‐existing heart failure, who were more than twice as likely to be classified as unavoidable in comparison to other health conditions (OR = 0.46, *p* < 0.0001). No significant differences were found for osteoskeletal muscle disorders, headaches, developmental/learning issues, or other miscellaneous diseases (Table 2).

**Table 2:** Comparison between avoidable ED and ED unavoidable visits (*N* = 43,437).

	Avoidable (*n* = 20,104)		Unavoidable (*n* = 23,333)			
Variables	*n*	%	*n*	%	*p*	Strength of association
Sex					< 0.0001^a^	1.40 (1.35–1.46)^a^
Female	9405	42.2	12,893	57.8		
Male	10,699	50.6	10,440	49.4		
Age (years)					< 0.0001^b^	−0.09^b^
< 15	3771	55.5	3011	44.5		
15–69	11,400	46.4	13,147	53.6		
≥ 70	4933	40.7	7175	59.3		
Access mode					< 0.0001^a^	0.17^c^
Ambulances	3232	31.3	7109	68.7		
GP referral	111	42.4	151	57.6		
Other structure	3	50.0	3	50.0		
Own vehicle	16,758	51.1	16,070	48.9		
Main pre‐existing disease						
Hypertension	1512	40.0	2264	60.0	< 0.0001^a^	0.73 (0.69–0.79)^a^
Heart failure	32	28.8	79	71.2	< 0.0001^a^	0.46 (0.30–0.69)^a^
Other cardiovascular diseases	746	31.4	1631	68.6	< 0.0001^a^	0.50 (0.46–0.55)^a^
Total cardiovascular diseases	2051	37.8	3378	62.2	< 0.0001^a^	0.67 (0.63–0.71)^a^
Obesity	31	24.6	95	75.4	< 0.0001^a^	0.37 (0.25–0.55)^a^
Diabetes	602	39.8	911	60.2	< 0.0001^a^	0.74 (0.67–0.82)^a^
Gestational diabetes	3	20.0	12	80.0	0.051^a^	0.28 (0.08–1.00)^a^
Endocrine and metabolic disorders (no diabetes)	394	37.1	669	62.9	< 0.0001^a^	0.66 (0.58–0.75)^a^
Total endocrine‐metabolic diseases	943	38.6	1500	61.4	< 0.0001^a^	0.72 (0.66–0.78)^a^
Neurological disorders	351	37.9	574	62.1	< 0.0001^a^	0.69 (0.60–0.79)^a^
Degenerative nervous diseases	300	39.0	469	61.0	< 0.0001^a^	0.72 (0.62–0.83)^a^
Total neurological diseases	618	38.8	974	61.2	< 0.0001^a^	0.73 (0.66–0.81)^a^
Tumor	492	35.1	909	64.9	< 0.0001^a^	0.60 (0.54–0.67)^a^
COPD	99	25.7	286	74.3	< 0.0001^a^	0.39 (0.31–0.49)^a^
Lung diseases (no COPD)	256	39.3	395	60.7	< 0.0001^a^	0.73 (0.62–0.86)^a^
Total respiratory diseases	355	34.3	681	65.7	< 0.0001^a^	0.60 (0.52–0.68)^a^
Gastrointestinal diseases	392	33.1	794	66.9	< 0.0001^a^	0.55 (0.49–0.62)^a^
Psychiatric diseases	338	39.9	509	60.1	< 0.0001^a^	0.75 (0.65–0.86)^a^
Urinary system and kidney diseases	194	33.6	384	66.4	< 0.0001^a^	0.57 (0.48–0.67)^a^
Articular diseases	144	45.6	172	54.4	0.639^a^	0.95 (0.76–1.18)^a^
Osteoskeletal muscle disorders (no articular diseases)	145	43.7	187	56.3	0.240^a^	0.88 (0.71–1.09)^a^
Total osteoskeletal‐muscle disorders	280	45.5	335	54.5	0.499^a^	0.95 (0.81–1.11)^a^
Contagious diseases	106	32.1	224	67.9	< 0.0001^a^	0.53 (0.42–0.67)^a^
Hematic diseases	107	39.8	162	60.2	0.020^a^	0.75 (0.58–0.95)^a^
Headache	53	39.0	83	61.0	0.065^a^	0.72 (0.51–1.02)^a^
Developmental and learning disorders	41	41.8	57	58.2	0.317^a^	0.81 (0.55–1.22)^a^
Other diseases	670	45.4	805	54.6	0.254^a^	0.94 (0.85–1.04)^a^
All ED visits	20,104	46.3	23,333	53.7		1.00

Abbreviations: CI, confidence interval; COPD, chronic obstructive pulmonary disease; ED, emergency department; GP, general practitioner; OR, odds ratio.

^a^ *χ* ^2^ test and ORs (with 95% CI).

^b^ Spearman's *ρ*.

^c^ Cramér's *V*.

## 4 Discussion

The general aim of the study was to identify which ED visits could have potentially been managed within primary and community care services in a scenario of full implementation of CHCs. This was achieved by analyzing data from S.S. Trinità Hospital of Borgomanero in Northern Italy, reviewing 43,437 ED visits recorded in 2022.

Criteria were established based on emergency‐level triage data, chief complaints, additional symptoms/signs, pre‐existing conditions, patient age, including the day and time of ED visits. Applying these criteria to all ED visits in 2022 showed that 46.3% could be classified as avoidable ED visits. However, this finding should not be interpreted as evidence that 46.3% of ED visits would necessarily have been prevented through the full implementation of CHCs. Rather, this proportion represents ED visits that, based on criteria, were retrospectively identified as manageable within primary and community care settings.

Our findings indicated that avoidable ED visits were significantly more prevalent among participants aged < 15 years than among older adults, with a marked disparity in the proportion of unavoidable visits across these age groups (*p* < 0.0001).

Avoidable ED visits were also more frequent among male patients and individuals without significant comorbidities. Common clinical presentations associated with avoidable ED visits included trauma or burns, fever, eye symptoms, urological and otolaryngological symptoms, lumbar pain, and various types of non‐specific pain.

We have also estimated the validity of our set of criteria, on the basis of the proportion of avoidable visits that resulted in a hospitalization or in death: this error rate was 6.4% and was primarily due to visits related to social issues, diagnostic evaluations, and psychomotor agitation. These visits resulted in hospitalization or death in 41.0%, 32.0%, and 25.0% of cases, respectively. These data underscore that many of these visits, particularly those driven by social issues, could be effectively managed through the establishment of a well‐structured PC network, thereby preventing undue strain on ED services, which are not the most appropriate setting for addressing such needs.

The high proportion of avoidable ED visits observed in our study is not unique to our Italian setting. Similar results have been reported in several studies [20, 21], where a considerable percentage of ED visits have been classified as avoidable or non‐urgent. Despite differences in healthcare system organization, the persistence of this issue across countries suggests common challenges in access to and utilization of PC services [22].

CHCs represent a significant innovation in the Italian healthcare system, designed to bring care closer to the community by integrating multidisciplinary teams and promoting proactive, preventive approaches. Specifically, CHCs serve as strategic proximity facilities that intercept health needs early and initiate timely care pathways, thereby reducing inappropriate ED use. Indeed, our results align with John et al. [23], who demonstrated significant improvements in health indicators including quality of life, frequency of depressive episodes, glycemic control, and hospital admissions among populations receiving care via CHCs compared to standard care.

It is also important to recognize that other factors, including intrinsic patient‐related ones, can contribute to ED utilization, even despite the existence of a PC network; these are, for example, perceived urgency, anxiety, or lack of knowledge about alternative care options. Moreover, issues related to general practitioners (GPs), including mistrust in GP care versus hospital care and systemic problems like long waiting lists, also influence patient decisions [24, 25]. Notably, recent Italian surveys indicate that nearly 60.0% of nonurgent ED visits occurred without prior contact with GPs [26]. To alleviate the burden on EDs, the existence of a CHC is essential, but it will be important to tackle these patient‐related factors [27, 28]. This ongoing discussion highlights the complexity of emergency healthcare management and emphasizes the necessity for comprehensive community‐based care systems [29].

Our findings emphasize the practical implications of integrated healthcare systems, such as CHCs collaborating with healthcare professionals. The high proportion of avoidable ED visits among individuals aged < 15 years suggests that pediatric primary care services should also be considered within the CHC model. In the Italian healthcare system, primary care pediatricians represent the main point of access to healthcare for children up to 14 years of age [30]. Their integration into multidisciplinary teams within CHCs could improve the management of non‐urgent pediatric conditions, enhance continuity and accessibility of care, and contribute to reducing unnecessary ED utilization in this population [31].

The integration of these services, in a scenario in which the CHCs are open 5 days a week, 12 h a day, could potentially manage up to 46.0% of ED visits by providing timely, targeted responses to nonurgent care needs, alleviating ED overcrowding, and improving chronic disease management [32]. Looking ahead, the implementation of 24‐h CHCs staffed by nursing professionals could further reduce demand for emergency and urgent care services by ensuring continuous and accessible management of nonurgent health issues [29, 33].

This study has some limitations. First, it was conducted within a single hospital, which may affect the generalizability of the results. However, this allowed us to obtain a timely snapshot of emergency room admissions in a medium size hospital that can be assumed to be similar to the most part of similar structures in Italy. Second, the criteria for classifying ED visits were developed using data exclusively from clinical records, which was routinely recorded by different nurses with varying quality. This implied careful data cleaning to minimize potential misclassification errors. Two researchers with expertise in family and community nursing independently conducted the categorization to ensure greater reliability. Third, our findings reflect the local healthcare system's specific characteristics, including a network of primary care services, general practitioners, social services, and nonprofit organizations. This may limit the applicability of the results to other contexts.

Despite these limitations, our study provides valuable insights into strengthening primary care services and improving the integration of healthcare delivery. We exclusively utilized triage data to identify avoidable ED visits manageable by CHCs. The primary objective was to determine whether inappropriate and redirectable visits could be identified upon admission based solely on patient information available at triage with a minimal margin of error. This approach proved valuable not only for identifying visits that could be averted through local, non‐urgent primary care management but also for quantifying the potential margin of error generated by interventions aimed at reducing inappropriate ED utilization. However, the high rate of non‐urgent ED visits highlights the need for targeted strategies to optimize resource allocation and enhance care delivery. The criteria we developed seem able to capture local healthcare dynamics and highlight the crucial role that CHCs staffed with FCNs could play. Despite the specificity of the Italian health system, we believe that these results are largely transferable in other countries experiencing a rapid ageing of the population.

## 5 Conclusions

This study represents a first attempt to estimate avoidable accesses to ED through a network of territorial services, such as CHCs. Our findings emphasize the critical need to strengthen PC services to better manage the growing demand for hospital services. To maximize the effectiveness of CHCs in local contexts, however, it is crucial to ensure that community‐based services are both accessible and effective. Moreover, enabling free and immediate access to these services without the need for appointments would allow for a faster response to non‐urgent healthcare needs, ultimately reducing the strain on EDs.

Future research should extend the application of this method to other regions where CHCs are effectively operating, to enable comparative analyses. Such comparisons would contribute to refining the criteria for identifying avoidable visits to the ED and evaluating their relevance and adaptability across the various models of care envisioned within primary healthcare.

## Author Contributions

**Silvia Caristia:** conceptualization, methodology, data curation, formal analysis, and writing – original draft. **Isabella Santomauro:** conceptualization, methodology, formal analysis, and writing – original draft. **Erika Bassi:** data curation and writing – review and editing. **Laura Ferraris:** conceptualization and data curation. **Alberto Dal Molin:** conceptualization, writing – review and editing and supervision. **Fabrizio Faggiano:** conceptualization, methodology, data curation, writing – review and editing, supervision, project administration.

## Ethics Statement

This study was approved by the Novara Interagency Ethics Committee on January 27, 2023 (protocol number: 86/CE). The study was conducted in accordance with the ethical principles of the Declaration of Helsinki (as revised in 2013) and adhered to the STROBE reporting guidelines. Administrative data were collected retrospectively, with permission from the Novara Local Health Authority.

## Consent

The Novara Interagency Ethics Committee granted a waiver of informed consent, as the study exclusively utilized retrospectively collected, de‐identified administrative data that precluded identification of individual participants. The data were treated confidentially and in accordance with the current legislation on the protection of personal data, Legislative Decree June 30, 2003, as amended by Legislative Decree August 10, 2018, No. 101 on “Provisions for the adaptation of national legislation to the provisions of Regulation (EU) 2016/679.”

## Conflicts of Interest

The authors declare no conflicts of interest.

## Supporting information


Supporting File 1

Supporting File 2

## Data Availability

The data sets generated and/or analyzed during the current study are not publicly available to protect the patient's privacy, but are available from the corresponding author on reasonable request.
