# Supplementary material for: Identifying Avoidable Emergency Department Visits and the Potential Role of Community Health Centers in Northern Italy: A Retrospective Observational Study
Source: Health Care Sci. 2026 Sep 25:10.1002/hcs2.70103. Online ahead of print. doi: 10.1002/hcs2.70103 (PMC13614882; doi:10.1002/hcs2.70103)
Supplement: Supplementary file 2 [file HCS2-9999-0-s001.docx]

| **Supplement Table S1**. Characteristics of ED visits in 2022 (*N* = 43,437): characteristics of presentations and pre-existing diseases. | | | | | | | |
| --- | --- | --- | --- | --- | --- | --- | --- |
| **Characteristics of presentations** | ***n*** | **%** | **Non-urgent codes**  **(*n* = 39,864)** | | **Urgent codes (*n* = 3573)** | | ***p*** |
|  |  |  | ***n*** | **%** | ***n*** | **%** |  |
| Trauma or burn | 13,048 | 30.0 | 12,056 | 92.4 | 992 | 7.6 | 0.002 |
| Abdominal pain | 4081 | 9.4 | 3852 | 94.4 | 229 | 5.6 | < 0.0001 |
| Obstetrical/gynecological symptoms | 3233 | 7.4 | 3163 | 97.8 | 70 | 2.2 | < 0.0001 |
| Fever | 3187 | 7.3 | 3060 | 96.0 | 127 | 4.0 | < 0.0001 |
| Dyspnea | 2029 | 4.7 | 1451 | 71.5 | 578 | 28.5 | < 0.0001 |
| Other pains | 1988 | 4.6 | 1937 | 97.4 | 51 | 2.6 | < 0.0001 |
| Thoracic pain | 1674 | 3.9 | 1447 | 86.4 | 227 | 13.6 | < 0.0001 |
| Other symptoms | 1477 | 3.4 | 1299 | 88.0 | 178 | 12.0 | < 0.0001 |
| Urological symptoms | 1399 | 3.2 | 1294 | 92.5 | 105 | 7.5 | 0.319 |
| Eye problems | 1317 | 3.0 | 1307 | 99.2 | 10 | 0.8 | < 0.0001 |
| Other nervous system symptoms | 1296 | 3.0 | 1008 | 77.8 | 288 | 22.2 | < 0.0001 |
| Hypertension/tachycardia/heart rate alterations | 1207 | 2.8 | 1031 | 85.4 | 176 | 14.6 | < 0.0001 |
| ENT symptoms | 1060 | 2.4 | 1051 | 99.1 | 9 | 0.9 | < 0.0001 |
| Nausea/vomit/diarrhea | 709 | 1.6 | 683 | 96.3 | 26 | 3.7 | < 0.0001 |
| Lumbar pain | 647 | 1.5 | 639 | 98.8 | 8 | 1.2 | < 0.0001 |
| Cough | 603 | 1.4 | 592 | 98.2 | 11 | 1.8 | < 0.0001 |
| Allergic reaction | 581 | 1.3 | 531 | 91.4 | 50 | 8.6 | 0.737 |
| Non-traumatic hemorrhage | 572 | 1.3 | 531 | 92.8 | 41 | 7.2 | 0.354 |
| Syncope or fainting | 556 | 1.3 | 497 | 89.4 | 59 | 10.6 | 0.040 |
| Tumefaction/mass/swelling | 520 | 1.2 | 512 | 98.5 | 8 | 1.5 | < 0.0001 |
| Psychomotor agitation | 489 | 1.1 | 405 | 82.8 | 84 | 17.2 | < 0.0001 |
| Edema | 434 | 1.0 | 422 | 97.2 | 12 | 2.8 | < 0.0001 |
| Headache | 434 | 1.0 | 425 | 97.9 | 9 | 2.1 | < 0.0001 |
| Referral for medical tests | 300 | 0.7 | 283 | 94.3 | 17 | 5.7 | 0.108 |
| Acute neurological symptoms | 229 | 0.5 | 120 | 52.4 | 109 | 47.6 | < 0.0001 |
| Intoxication/poisoning/drug abuse | 204 | 0.5 | 155 | 76.0 | 49 | 24.0 | < 0.0001 |
| Metabolic symptoms | 62 | 0.1 | 55 | 88.7 | 7 | 11.3 | 0.382 |
| Social problems | 53 | 0.1 | 53 | 100.0 | 0 | 0.0 | — |
| Shock | 36 | 0.1 | 4 | 11.1 | 32 | 88.9 | < 0.0001 |
| Coma | 12 | 0.0 | 1 | 8.3 | 11 | 91.7 | < 0.0001 |
| **Main pre-existing diseases** |  |  |  |  |  |  |  |
| Hypertension | 3776 | 8.7 | 3373 | 89.3 | 403 | 10.7 | < 0.0001 |
| Heart failure | 111 | 0.3 | 97 | 87.4 | 14 | 12.6 | 0.058 |
| Other cardiovascular diseases | 2377 | 5.5 | 2015 | 84.8 | 362 | 15.2 | < 0.0001 |
| Total cardiovascular diseases (with hypertension)^a^ | 5429 | 12.5 | 4784 | 88.1 | 645 | 11.9 | < 0.0001 |
| Obese | 126 | 0.3 | 112 | 88.9 | 14 | 11.1 | 0.160 |
| Diabetes | 1513 | 3.7 | 1318 | 87.1 | 195 | 12.9 | < 0.0001 |
| Gestational diabetes | 15 | 0.0 | 14 | 93.3 | 1 | 6.7 | 0.875 |
| Endocrine and metabolic disorders (no diabetes) | 1063 | 2.6 | 957 | 90.0 | 106 | 10.0 | 0.006 |
| Total endocrine-metabolic diseases (no obeses)^a^ | 2443 | 5.6 | 2162 | 88.5 | 281 | 11.5 | < 0.0001 |
| Neurological disorders | 925 | 2.1 | 782 | 84.5 | 143 | 15.5 | < 0.0001 |
| Degenerative nervous diseases | 769 | 1.8 | 639 | 83.1 | 130 | 16.9 | < 0.0001 |
| Total neurological diseases^a^ | 1592 | 3.7 | 1340 | 84.2 | 252 | 15.8 | < 0.0001 |
| Tumor | 1401 | 3.2 | 1227 | 87.6 | 174 | 12.4 | < 0.0001 |
| COPD | 385 | 0.9 | 321 | 83.4 | 64 | 16.6 | < 0.0001 |
| Lung diseases (no COPD) | 651 | 1.5 | 602 | 92.5 | 49 | 7.5 | 0.830 |
| Total respiratory diseases^a^ | 1036 | 2.4 | 923 | 89.1 | 113 | 10.9 | 0.002 |
| Gastrointestinal diseases | 1186 | 2.9 | 1092 | 92.1 | 94 | 7.9 | 0.819 |
| Psychiatric diseases | 847 | 2.1 | 746 | 88.1 | 101 | 11.9 | < 0.0001 |
| Urinary system and kidney diseases | 578 | 1.4 | 489 | 84.6 | 89 | 15.4 | < 0.0001 |
| Articular diseases | 316 | 0.8 | 290 | 91.8 | 26 | 8.2 | 0.750 |
| Osteoskeletal muscle disorders (no articular diseases) | 332 | 0.8 | 294 | 88.5 | 38 | 11.5 | 0.012 |
| Total osteoskeletal-muscle disorders^a^ | 615 | 1.5 | 556 | 90.4 | 59 | 0.6 | 0.086 |
| Contagious diseases | 330 | 0.8 | 280 | 84.9 | 50 | 15.1 | < 0.0001 |
| Hematic diseases | 269 | 0.7 | 240 | 89.2 | 29 | 10.8 | 0.064 |
| Headache | 136 | 0.3 | 127 | 93.4 | 9 | 6.6 | 0.621 |
| Developmental and learning disorders | 98 | 0.2 | 90 | 91.8 | 8 | 8.2 | 0.878 |
| Other diseases | 1,475 | 3.6 | 1,326 | 89.9 | 149 | 10.1 | 0.001 |

*Note*: ^a^The total of accesses with one or more pre-existing diseases, considering also patients with more of a characteristic of presentation.

Abbreviations: COPD, chronic obstructive pulmonary disease; ED, emergency department; ENT, ear, nose, and throat.

| **Supplement Table S2**. Characteristics of avoidable ED visits by type of discharge (home *vs.* hospitalization/death): characteristics of presentation and pre-existing diseases. | | | | | | | |
| --- | --- | --- | --- | --- | --- | --- | --- |
| **Characteristics of presentation** | **Avoidable ED visits (*n* = 20,104)** | | **Home discharge**  **(*n* = 18,813)** | | **Hospitalization/death (*n* = 1291)** | | ***p*^a^** |
|  | ***n*** | **%^b^** | ***n*** | **%^c^** | ***n*** | **%^c^** |  |
| Trauma or burn | 9150 | 45.5 | 8778 | 95.9 | 372 | 4.1 | < 0.0001 |
| Fever | 1635 | 8.1 | 1445 | 88.4 | 190 | 11.6 | < 0.0001 |
| Other pains | 1520 | 7.6 | 1461 | 96.1 | 59 | 3.9 | < 0.0001 |
| Eye problems | 1160 | 5.8 | 1154 | 99.5 | 6 | 0.5 | < 0.0001 |
| Other symptoms | 933 | 4.6 | 787 | 84.3 | 146 | 15.7 | < 0.0001 |
| Urological symptoms | 824 | 4.1 | 760 | 92.2 | 64 | 7.8 | 0.108 |
| ENT symptoms | 722 | 3.6 | 707 | 97.9 | 15 | 2.1 | < 0.0001 |
| Lumbar pain | 507 | 2.5 | 488 | 96.3 | 19 | 4.7 | 0.014 |
| Nausea/vomit/diarrhea | 418 | 2.1 | 356 | 85.1 | 62 | 14.9 | < 0.0001 |
| Tumefaction/mass/swelling | 412 | 2.0 | 395 | 95.9 | 17 | 4.1 | 0.057 |
| Cough | 380 | 1.9 | 347 | 91.3 | 33 | 8.7 | 0.071 |
| Allergic reaction | 352 | 1.8 | 343 | 97.4 | 9 | 2.6 | 0.004 |
| Edema | 342 | 1.7 | 311 | 90.9 | 31 | 9.1 | 0.046 |
| Obstetrical/gynecological symptoms | 332 | 1.7 | 313 | 94.3 | 19 | 5.7 | 0.601 |
| Nontraumatic hemorrhage | 316 | 1.6 | 280 | 88.6 | 36 | 11.4 | < 0.0001 |
| Psychomotor agitation | 264 | 1.3 | 198 | 75.0 | 66 | 25.0 | < 0.0001 |
| Syncope or fainting | 197 | 1.0 | 175 | 88.8 | 22 | 11.2 | 0.007 |
| Intoxication/poisoning/drug abuse | 80 | 0.4 | 66 | 82.5 | 14 | 17.5 | < 0.0001 |
| Other nervous system symptoms | 70 | 0.3 | 61 | 87.1 | 9 | 12.9 | 0.032 |
| Abdominal pain^d^ | 84 | 0.4 | 80 | 95.2 | 4 | 4.8 | 0.536 |
| Headache | 56 | 0.3 | 53 | 94.6 | 3 | 5.4 | 0.061 |
| Social problems | 27 | 0.1 | 16 | 59.3 | 11 | 40.7 | < 0.0001 |
| Dyspnea | 25 | 0.1 | 21 | 84.0 | 4 | 16.0 | 0.061 |
| Thoracic pain^d^ | 30 | 0.1 | 29 | 96.7 | 1 | 3.3 | 0.498 |
| Hypertension/tachycardia/heart rate alterations | 14 | 0.1 | 14 | 100.0 | 0 | 0.0 | — |
| Metabolic symptoms | 5 | 0.0 | 5 | 100.0 | 0 | 0.0 | — |
| Referral for medical verifications^e^ | 249 | 1.2 | 170 | 68.3 | 79 | 31.7 | < 0.0001 |

*Note*: ^a^Univariate logistic regression: Chi-squared test with 95% confidence interval. ^b^Applied to all accesses (*N* = 43,437). ^c^Row percentage (based on the total for each major problem). ^d^White codes at triage. ^e^This access problem of access primarily consisted of planned prehospital admissions and other medical tests required by GPs or other hospital wards.

Abbreviations: ED, emergency department; ENT, ear, nose, and throat; GP, general practitioner.

| **Supplement Table S3**. Avoidable and unavoidable accesses by outcome at discharge and the magnitude of the error. | | | |
| --- | --- | --- | --- |
|  | **Avoidable accesses** | | |
| **Type of discharge** | **No** | **Yes** | **Total type of discharge** |
| Home discharge | 18,508 (79.3%) | 18,813 (93.6%) | 37,321 (85.9%) |
| Hospitalization/death | 4825 (20.7%) | 1291 (6.4%) | 6116 (14.1%) |
| **Total** | 23,333 (53.7%) | 20,104 (46.3%) | 43,437 (100.0%) |

| **Supplement Table S4.** Characteristics of avoidable ED visits (*N* = 20,104) by type of discharge (home *vs.* hospitalization/death): sex, age, entry time, day of trading, access mode, pre-existing diseases. | | | | | | |
| --- | --- | --- | --- | --- | --- | --- |
| **Variables** | **Home discharge**  **(*n* = 18,813)** | | **Hospitalization/**  **death**  **(*n* = 1,291)** | | ***p*** | **Strength of association** |
|  | ***n*** | **%** | ***n*** | **%** |  |  |
| **Sex** |  |  |  |  | 0.006^a^ | 0.85 (0.76–0.95)^a^ |
| Female | 8753 | 93.1 | 652 | 6.9 |  |  |
| Male | 10,060 | 94.0 | 639 | 6.0 |  |  |
| **Age (years)** |  |  |  |  | < 0.0001^b^ | 0.15^b^ |
| < 15 | 3619 | 96.0 | 152 | 4.0 |  |  |
| 15–69 | 10,951 | 96.1 | 449 | 3.9 |  |  |
| ≥ 70 | 4243 | 86.0 | 690 | 13.0 |  |  |
| **Entry time (triage hour)** |  |  |  |  | < 0.0001^a^ | 4.94 (2.45–9.95)^a^ |
| Night | 562 | 98.6 | 8 | 1.4 |  |  |
| Day | 18,251 | 93.4 | 1283 | 6.6 |  |  |
| **Day of entry** |  |  |  |  | 0.011^a^ | 1.18 (1.04–1.34)^a^ |
| Weekend | 5548 | 94.3 | 338 | 5.7 |  |  |
| Weekday | 13,265 | 93.3 | 953 | 6.7 |  |  |
| **Access mode** |  |  |  |  | < 0.0001^a^ | 0.26^c^ |
| Ambulances | 2641 | 81.7 | 591 | 18.3 |  |  |
| GPs’ referral | 90 | 81.1 | 21 | 18.9 |  |  |
| Other structure | 2 | 66.7 | 1 | 33.3 |  |  |
| Own vehicle | 16,080 | 95.9 | 678 | 4.1 |  |  |
| **Main pre-existing diseases** | | | | | | |
| Hypertension | 1387 | 91.7 | 125 | 8.3 | < 0.0001^a^ | 1.56 (1.29–1.90)^a^ |
| Heart failure | 22 | 68.7 | 10 | 31.3 | < 0.0001^a^ | 7.60 (3.59–16.08)^a^ |
| Other cardiovascular diseases | 645 | 86.5 | 101 | 13.5 | < 0.0001^a^ | 2.76 (2.21–3.43)^a^ |
| Obesity | 24 | 77.4 | 7 | 22.6 | < 0.0001^a^ | 4.86 (2.09–11.31)^a^ |
| Diabetes | 539 | 89.5 | 63 | 10.5 | < 0.0001^a^ | 1.99 (1.53–2.61)^a^ |
| Gestational diabetes | 2 | 66.7 | 1 | 33.3 | 0.084^a^ | 8.29 (0.75–91.58)^a^ |
| Endocrine and metabolic disorders  (no diabetes) | 355 | 90.1 | 39 | 9.9 | < 0.0001^a^ | 1.85 (1.32–2.59)^a^ |
| Neurological disorders | 307 | 87.5 | 44 | 12.5 | < 0.0001^a^ | 2.43 (1.76–3.36)^a^ |
| Degenerative nervous diseases | 253 | 84.3 | 47 | 15.7 | < 0.0001^a^ | 3.17 (2.31–4.36)^a^ |
| Tumor | 423 | 86.0 | 69 | 14.0 | < 0.0001^a^ | 2.82 (2.17–3.66)^a^ |
| COPD | 79 | 79.8 | 20 | 20.2 | < 0.0001^a^ | 4.26 (2.60–6.98)^a^ |
| Lung diseases (no COPD) | 238 | 93.0 | 18 | 7.0 | 0.351^a^ | 1.26 (0.78–2.04)^a^ |
| Gastrointestinal diseases | 338 | 86.2 | 54 | 13.8 | < 0.0001^a^ | 2.73 (2.04–3.67)^a^ |
| Psychiatric diseases | 296 | 87.6 | 42 | 12.4 | < 0.0001^a^ | 2.41 (1.73–3.34)^a^ |
| Urinary system and kidney diseases | 152 | 78.3 | 42 | 21.7 | < 0.0001^a^ | 4.72 (3.34–6.69)^a^ |
| Articular diseases | 131 | 91.0 | 13 | 9.0 | 0.086^a^ | 1.65 (0.93–2.93)^a^ |
| Osteoskeletal muscle disorders  (no articular diseases) | 129 | 89.0 | 16 | 11.0 | 0.006^a^ | 2.07 (1.23–3.50)^a^ |
| Contagious diseases | 91 | 85.9 | 15 | 14.1 | < 0.0001^a^ | 2.76 (1.59–4.78)^a^ |
| Hematic diseases | 89 | 83.2 | 18 | 16.8 | < 0.0001^a^ | 3.39 (2.04–5.65)^a^ |
| Headache | 50 | 94.3 | 3 | 5.7 | 0.993^a^ | 0.99 (0.31–3.19)^a^ |
| Developmental and learning disorders | 40 | 97.6 | 1 | 2.4 | 0.369^a^ | 0.41 (0.06–3.01)^a^ |
| Other diseases | 615 | 91.8 | 55 | 8.2 | 0.004^a^ | 1.51 (1.14–2.00)^a^ |
| All ED visits | 20,104 | 46.3 | 23,333 | 53.7 |  | 1.00 |

*Note*: ^a^Chi-squared test and ORs (95% CI). ^b^Spearman’s Rho. ^c^Cramér’s V.

Abbreviations: CI, confidence interval; COPD, chronic obstructive pulmonary disease; ED, emergency department; GP, general practitioner; OR, odds ratio.
